# Return-to-work rates and predictors of absence duration after COVID-19 over the course of the pandemic

**DOI:** 10.5271/sjweh.4077

**Published:** 2023-03-30

**Authors:** Bart Aben, Robin N Kok, Astrid de Wind

**Affiliations:** 1 HumanTotalCare BV, Department of Research and Development, Zwarte Woud 10, Utrecht, The Netherlands; 2 Amsterdam UMC location University of Amsterdam, Public and Occupational Health, Meibergdreef 9, Amsterdam, The Netherlands; 3 Amsterdam Public Health, Societal Participation and Health, Amsterdam, The Netherlands

**Keywords:** Cox proportional hazards model, long COVID, occupational health, return to work, RTW, SARS-CoV-2, sick leave, sickness absence, survival analysis

## Abstract

**Objectives:**

The aim of this study was to evaluate employee return-to-work (RTW) rates and examine predictors of absence duration after COVID-19. RTW rates were referenced against RTW rates after absence due to flu-like symptoms and assessed over the course of the pandemic.

**Methods:**

Routinely collected data from a nationally operating Dutch occupational health service was used. The data were retrieved from employees who reported sick due to COVID-19 (N=30 396) or flu-like symptoms (N=15 862). Data consisted of responses to a triage survey combined with longitudinal register-based information on sickness absence. RTW rates after COVID-19 were evaluated through Kaplan-Meier estimates and compared to RTW rates for flu-like symptoms, and between three periods with different dominant virus variants. Predictors for absence duration were examined through Cox proportional hazards models.

**Results:**

RTW after COVID-19 was found to be notably later than after flu-like symptoms (median RTW=10 versus 6 days, respectively). On average, 5.5% of employees who contracted COVID-19 were absent for over 12 weeks. Time-to-RTW shortened as different virus variants became dominant over time. The main predictors contributing to later RTW were older age, female sex, belonging to a risk group, and the symptoms shortness of breath and fatigue.

**Conclusions:**

Estimates of the RTW rate after COVID-19 and identification of predictors may aid healthcare professionals in gaining insight into variations in the disease course and rehabilitation process. The present findings can help employers and policy-makers grasp the impact of COVID-19 on the workplace.

COVID-19, the disease caused by infection with the SARS-CoV-2 virus, has an immense impact on the world (1, 2). Apart from highly visible effects in the public health domain (eg, excess mortality), the effects on the working population have been large and diverse. Some companies have been able to flexibly adapt to prevailing conditions to minimize disease transmission and retain productivity, while others have been affected greatly by high infection rates and governmental interventions, such as lockdowns and mandatory closures. Regardless of scale, all workers have been affected in some way by the pandemic, leading to a sizeable impact on occupational health.

In The Netherlands, the detection of ‘patient zero’ on 27 February 2020 initiated a sequence of governmental measures. In early March 2020, the Dutch government advised working from home as part of a partial lockdown. This lockdown was relaxed in June 2020 when testing became widely available, until increased infection rates necessitated a new lockdown from October 2020 to June 2021. As vaccines became available from January 2021, in August 2021 approximately 85% of Dutch adults had received an initial dose, and 77% were fully vaccinated (3). In November 2021, worries about the new, more infectious omicron variant in The Netherlands led to new restrictions. In the following months, this new lockdown was gradually relaxed until measures were almost completely abolished in April 2022 (4).

At the start of the pandemic, little was known about symptoms and return-to-work (RTW) patterns among COVID-19 patients, leading to a considerable amount of uncertainty as to the prognosis and impact of the pandemic for employers and employees. As time progressed, some studies identified tentative risk factors for slower RTW after COVID-19, such as illness severity (eg, hospitalization), comorbidity, older age, and female sex (5–8). Population-wide dominance of successive COVID-19 variants (eg, alpha, delta, omicron), behavioral factors (eg, risk averseness in workers), precautionary governmental measures (eg, lockdowns, working from home advice) and employers (eg, personal protective equipment) are also likely to have affected RTW. Although the emergence of long COVID (9) has shown that long-term sickness absence potentially occurs in a sizeable proportion of the workforce (6), little information is available on symptoms and the exact course of absence trajectories throughout the pandemic.

The current study used data from a nationally operating Dutch occupational health service (OHS) collected over the course of two years of COVID-19. The primary aim of this study was to evaluate RTW rates after COVID-19 as compared to RTW rates after flu-like symptoms and in relation to successive dominant COVID-19 variants. The secondary aim of this study was to explore various predictors of time-to-RTW after COVID-19, specifically those related to worse outcomes.

## Method

### Design

This study used routinely collected data from a nationally operating Dutch OHS (ArboNed). The OHS mainly covers small and medium-sized enterprises (SMEs) with ≤250 employees. It is represented in all business activities of the Dutch labor market but mostly in industry and energy, followed by commercial services and non-commercial services.

The data consisted of responses to a web-based triage survey combined with longitudinal register-based information on sickness absence. As part of routine service, employees served by the OHS are asked to complete a 5–15 minute triage survey shortly after reporting sick [time of completion after sick leave notification: median 4 (interquartile rate 2–7) days]. Medical professionals use the survey responses to assess the employee’s health status and expected absence duration. The survey covers a range of topics, such as self-reported cause of absence, symptoms, job satisfaction and lifestyle. While the exact response rate for the current survey is unknown, generally at least 65% of the invited employees respond. This response rate is obtained by dividing the number of people who completed the survey by the number of people who should have received the survey. In some cases, contact details of the employee may have been incorrect or missing. For administrative reasons, it is unknown how many of these employees did not receive the survey. Since the number of people who should have received the survey is larger than the number of people that actually received the survey, the reported response rate is likely an underestimation of the actual response rate.

On 19 March 2020, shortly after the COVID-19 pandemic started in The Netherlands, questions related to COVID-19 were added to the triage survey. These questions included self-reported infection status, COVID-19-specific disease symptoms, disease burden, and COVID-19-specific risk factors as understood at that time. The questions from the triage survey that were used for the current study are listed in supplementary material (www.sjweh.fi/article/4077) figure S1. The data from the triage survey were supplemented with data retrieved from the data-registry of the OHS, including demographic information, first day of absence, absence status at the end of the follow-up period (eg, sick, recovered, deceased, or reason for loss to follow-up such as end of contract), and – if recovered – time-to-RTW. As required by law, this data is provided by the employer of the sick employee through a standardized and automated digital registration process. The dataset was fully anonymized by removing all personal identifiers before it was made available to the researchers. As the data were routinely collected as part of the OHS’s care provision, approval of a medical ethics committee was not sought.

### Study population

This study initially included employees aged ≥18 years who reported sick between 19 March 2020 and 18 March 2022, completed the triage survey, and reported COVID-19 (N=31 103) or flu-like symptoms (N=16 225) as a reason for reporting sick. The sample with flu-like symptoms served as a reference for RTW analyses and only included employees who confirmed not being infected with COVID-19. Absence was defined as being the result of COVID-19 when employees reported having tested positive for COVID-19 (including self-tests), or reported a COVID-19 diagnosis confirmed by a doctor or Municipal Health Service. Survey data were only used from participants who consented to the use of their anonymized data for scientific research on the effect of COVID-19 on absenteeism. Follow-up on the absence status of these employees was obtained until 24 October 2022.

### Variables

The outcome variable ‘time-to-RTW’ was defined as the number of days between initial sick report and full recovery (ie, absence duration).

Dominance of virus variant was established based on pathogen surveillance by the National Institute for Public Health and the Environment (RIVM) in The Netherlands (10). The virus variant responsible for >50% of infections at a given time was considered dominant. This split encompassed observations with first day of absence between 15 February and 27 June 2021 (alpha dominant); 28 June and 27 December 2021 (delta dominant); and 28 December 2021 and 18 March 2022 (omicron dominant).

The following predictors were derived from the triage survey: disease burden (1–10, where 1 is no burden); risk group (no, perhaps/somewhat or yes; dummy coded); COVID-19 relevant symptoms of fatigue, headache, coughing, mild rhinitis, muscle pain, fever, loss of taste, sore throat, shortness of breath, coughing up mucus, nausea/vomiting, intestinal problems and no complaints (dummy coded to 0=not present, 1=present); job satisfaction (“I really like my work”; 1–7; 1=never, 7=always; treated as ordinal variable); and sleep disturbance (“Have you had trouble falling asleep in the past month?”; 1–5; 1=never, 5=always; treated as ordinal variable). The variable risk group was not subdivided in order to keep the number of predictors manageable.

**Figure 1:**
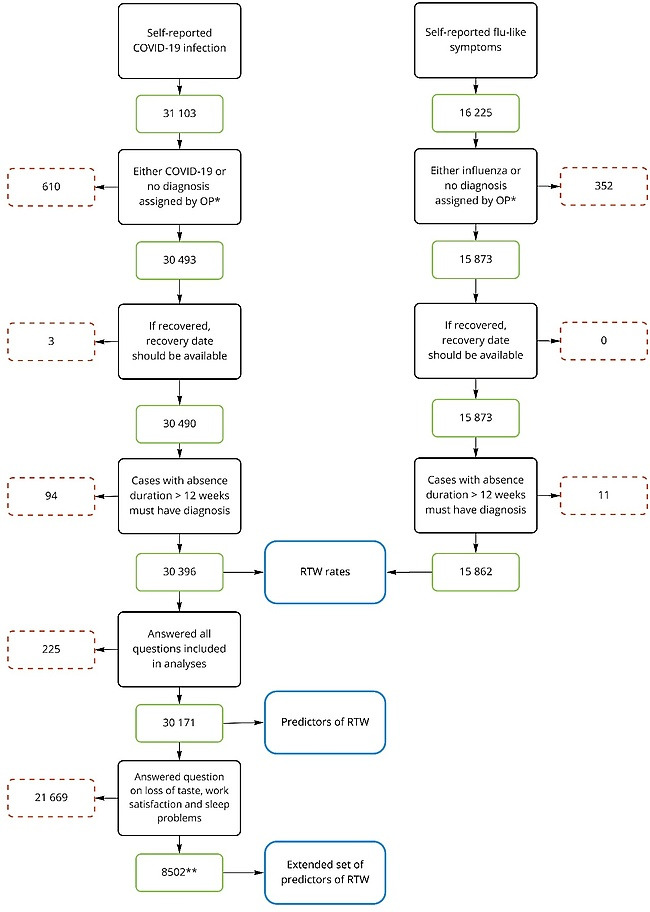
Flowchart of study inclusion criteria. * Employees that report sick usually only consult the occupational physician (OP) after six weeks of absence. Most cases with COVID-19 or flu-like symptoms will therefore not have a diagnosis assigned by the OP. Cases with self-reported COVID-19 or flu-like symptoms that were assigned a different diagnosis upon consultation with the OP were excluded. ** These participants had first day of absence between January 25, 2021 and March 18, 2022 and a self-estimated RTW of more than a week

As the triage survey used conditional questions to minimize respondent burden, some topics were omitted from the survey, depending on the employee’s personal situation. For example, the questions on job satisfaction and sleep disturbance were only answered by respondents who estimated their absence would take at least one week. The symptom ‘loss of taste’ was not included in the triage survey until 25 January 2021, as up to then there was no consensus on whether loss of taste was a symptom of COVID-19 (11). To account for these differences, separate analyses were performed on the initial data set and on a subset of the data that contained an extended set of predictors. This is visualized in figure 1.

### Data-analysis

Descriptive statistics were used to summarize the study population. Participants lost to follow-up were censored and classified as sick until the day of loss to follow-up. They made no contribution to estimations beyond that time point.

In line with the first aim of the study, RTW rates after COVID-19 were evaluated through Kaplan-Meier estimates (ie, survival functions) that estimated the probability of RTW as a function of absence duration. First, the survival function for COVID-19 was estimated and compared to that of flu-like symptoms using a log-rank test. Second, the COVID-19 sample was split into three subsamples based on dominant virus variant on the first day of absence. Survival functions were estimated and multivariate log-rank tests were used to test for differences between the survival distributions of the three subsamples, followed by Bonferroni-corrected pairwise log-rank tests. This analysis was repeated after matching samples on age and sex using propensity logit scores, with a 1:1 ratio and no caliper. The sample of participants with flu-like symptoms was matched to the total sample of COVID-19 participants. The delta- and omicron-dominant samples were matched to the alpha-dominant sample.

In line with the second aim of the study, predictors of time-to-RTW after COVID-19 were identified through univariate and multivariate Cox proportional hazards (CPH) models. In the CPH model, hazard ratios (HR) are calculated to evaluate the relationship between predictors and time-to-RTW (ie, absence duration). The HR can be interpreted as a relative risk (RR) of returning to work associated with a certain predictor while holding the other predictors in the model constant. HR=1 indicates no difference in time-to-RTW for different levels of a predictor. HR<1 indicates that a higher predictor value delays RTW (ie, decreases the probability of returning to work at a given time). HR >1 indicates that a higher predictor value accelerates RTW (ie, increases the probability of returning to work at a given time).

The concordance index is reported as a measure of goodness-of-fit of the multivariate CPH models. The assumption of proportional hazards was met, as evaluated through visual inspection of scaled Schoenfeld residuals.

To make maximum use of available data, two predictive analyses were performed. The following predictors were included in the first analysis: age; sex; risk group; disease burden; and the symptoms fatigue, headache, coughing, mild rhinitis, muscle pain, fever, sore throat, shortness of breath, coughing up mucus, nausea/vomiting, intestinal problems and no complaints. First, univariate models were fit, followed by a multivariate CPH model that included all listed predictors.

A second predictive analysis was performed on a smaller subset of the data but with an extended set of predictors. In addition, this analysis included the symptom ‘loss of taste’. The questions on job satisfaction and sleep disturbance – which were only available for cases with an estimated absence duration of over a week – were also added. Since responses with any missing value were excluded from the model, the data entered into the second CPH model comprises data collected after addition of the symptom ‘loss of taste’ to the survey (25 January 2021) and cases with an estimated absence duration of a week or more (figure 1). We deemed this shortcoming acceptable in comparison with the added value of the three additional predictors. Again, univariate CPH models were fit first, followed by a multivariate CPH model.

The predictor ‘disease burden’ was omitted from the final multivariate models for reasons of multicollinearity (VIF=10.2).

Kaplan-Meier and CPH analyses were performed using the lifelines 0.26.3 package (12) in Python 3.8.

## Results

### Participants

A total of 31 103 employees reported COVID-19 on the triage survey. After applying inclusion criteria (figure 1), 30 396 participants remained (table 1). Of these participants, 1319 were censored as they did not recover within the follow-up period (1080), their contract with the employer ended (137), their employer’s contract with the OHS ended (92), they were deceased (4), or they entered disability benefits programs (6).

**Table 1:** Participant characteristics. [SD=standard deviation; COPD=chronic obstructive pulmonary disease.]

Characteristics	Analysis of RTW rate ^a^			Analysis of predictors of time to RTW ^b^			Analysis of extended set of predictors of time to RTW ^c^		
		
	N	%	Mean (SD)	N	%	Mean (SD)	N	%	Mean (SD)
Total	30 396	100		30 171	100		8502	100	
Age (years)			40.4 (12.0)			40.4 (12.0)			42.9 (11.9)
Sex									
Male	15 352	50.5		15 215	50.4		4071	47.9	
Female	15 029	49.5		14 956	49.6		4431	52.1	
Prefer not to say	15	0.0							
Disease burden (1–10)			6.2 (2.4)			6.2 (2.4)			6.9 (2.1)
Risk group									
No	24 301	79.9		24 229	80.3		6394	75.2	
Perhaps/somewhat	3000	9.9		2951	9.8		1046	12.3	
Yes ^d^	2961	9.7		2991	9.9		1062	12.5	
Respiratory disorder (asthma, COPD)	2120	36.1		2110	36.0		740	35.5	
Age	1297	22.1		1295	22.1		501	24.0	
Chronical disease	903	15.4		900	15.4		340	16.3	
Cardiovascular disease	734	12.5		732	12.5		270	13.0	
Diabetes	590	10.0		590	10.1		224	10.7	
Obesity	586	10.0		584	10.0		228	10.9	
Compromised immune system due to disease and/or medication	472	8.0		469	8.0		168	8.1	
Smoking	228	4.9		285	4.9		113	5.4	
Unknown	134	0.4							
Symptoms ^d^									
Fatigue	22 785	75.2		22 697	75.2		7134	83.9	
Coughing	22 153	73.1		22 066	73.1		6688	78.7	
Headache	21 619	71.4		21 531	71.4		6469	76.1	
Mild rhinitis	18 211	60.1		18 148	60.2		5254	61.8	
Muscle pain	16 919	55.8		16 849	55.8		5285	62.2	
Sore throat	15 337	50.6		15 280	50.6		4283	50.4	
Fever	14 691	48.5		14 625	48.5		4521	53.2	
Coughing up mucus	10 619	35.0		10 589	35.1		3567	42.0	
Shortness of breath	10 102	33.3		10 061	33.3		3524	41.4	
Loss of taste	6728	26.9					3148	37.0	
Nausea/vomiting	4401	14.5		4384	14.5		1647	19.4	
Intestinal problems	3849	12.7		3840	12.7		1354	15.9	
No complaints	776	2.6		773	2.6		65	0.8	
Unknown	97	0.3							
Job satisfaction ^e^									5.8 (1.1)
Sleep disturbance ^e^									1.9 (0.9)

a Employees with COVID-19, irrespective of full completion of the survey. This sample was used to obtain general RTW rates that were compared to RTW for flu-like symptoms.

b Employees with COVID-19 who fully completed the survey.

c Employees who fully completed the survey, including the later added questions on loss of taste, job satisfaction, and sleep problems added later.

d Multiple answers possible.

e This question was answered only by employees with first day of absence between 25 January 2021 and 18 March 2022 and a self-estimated RTW of >1 week.

A total of 16 225 employees reported sick on the triage survey because of flu-like symptoms. After applying inclusion criteria (figure 1), 15 862 participants remained (table 1). Of these participants, 413 were censored as they did not recover within the follow-up period (369), their contract with the employer ended (27), or their employer’s contract with the OHS ended (17).

### RTW rate analyses

**Figure 2:**
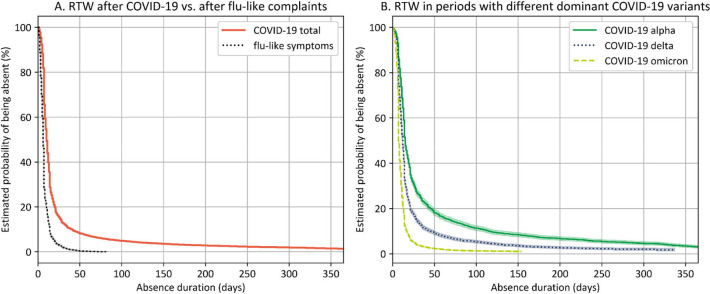
A) Survival curve of employees with self-reported COVID-19 between 19 March 2020 and 18 March 2022 (COVID-19 total), referenced against employees with self-reported flu-like symptoms in the same time period. B) Survival curves of employees with self-reported COVID-19 in time periods with different dominant variants. Variant dominance information was taken from the Dutch National Institute for Public Health and the Environment (RIVM; coronadashboard.government.nl/landelijk/varianten). Error bands represent exponential Greenwood confidence intervals.

The survival curve for absence ≤365 days after self-reported COVID-19 is displayed in figure 2A. As a reference, the survival curve for respondents who reported sick with flu-like symptoms is also displayed (see supplementary table S1 for characteristics of the sample). The survival rate differed between the two samples, χ^2^ (1)=7294.57, P*<*0.001. Median time-to-RTW after COVID-19 was after 10 compared to 6 days for flu-like symptoms. Table 2 lists the probabilities of returning to work after COVID-19 at various absence durations.

**Table 2:** Probability of returning to work after COVID-19 at various return-to-work (RTW) rates and in time periods with different dominant virus variants. The sample for flu-like symptoms and the total COVID-19 sample include observations with first day of absence between 19 March 2020 and 18 March 2022. The other samples include observation with first day of absence between 15 February 2021 and 27 June 2021 (alpha dominant), 28 June 2021 and 27 December 2021 (delta dominant), and 28 December 2021 and 18 March 2022 (omicron dominant). [CI=confidence interval]

	Flu-like symptoms		COVID-19							

			Total		Alpha dominant		Delta dominant		Omicron dominant	
				
	% (95% CI)	Median (95% CI)	% (95% CI)	Median (95% CI)	% (95% CI)	Median (95% CI)	% (95% CI)	Median (95% CI)	% (95% CI)	Median (95% CI)
Time-to-RTW (days)		6 (6 -6)		10 (10 -10)		15 (14 -15)		12 (12 -12)		7 (7 -8)
RTW (weeks)										
≤1	71.6 (70.9–72.3)		34.4 (33.8–34.9)		14.0 (12.9–15.2)		25.2 (24.1–26.3)		50.7 (49.9–51.5)	
≤3	91.9 (91.4–92.3)		71.2 (70.7–71.8)		48.5 (46.9–50.1)		67.5 (66.3– 68.8)		87.5 (87.0– 88.1)	
≤6	99.2 (99.0–99.4)		90.6 (90.3–90.9)		79.2 (77.9– 80.5)		89.6 (88.8– 90.4)		97.3 (97.0–97.5)	
≤12	100.0 (100.0–100.0)		94.5 (94.2–94.7)		87.2 (86.0– 88.2)		94.2 (93.5, 94.8)		98.6 (98.4–98.8)	
≤26	100.0 (100.0–100.0)		97.0 (96.8– 97.2)		92.9 (92.0–93.7)		97.1 (96.6–97.5)		*	

* There were no omicron-infected employees with RTW after six months at the time of writing this report.

To evaluate changes in RTW throughout the pandemic, the data were split into three subsamples depending on which virus variant was dominant on the first day of absence (supplementary table S1). Multivariate log-rank tests showed a difference between the three survival distributions, χ^2^ (2)=3389.03, P*<*0.001 (figure 2B). Pairwise comparisons showed differences between all three distributions (P<0.001). These results indicate that RTW was latest when the alpha variant was dominant (median=15 days) and became earlier as the delta (median=12 days) and omicron variant (median=7 days) emerged (table 2). In the alpha-dominant period, 12.8% returned to work after 12 weeks, compared to 5.8% in the delta-dominant period and 1.4% in the omicron-dominant period.

These analyses were repeated after propensity score matching of the samples. This achieved samples that were better balanced in regard to age and sex. Results are displayed in supplementary table S2 and figure S2. After balancing, the results remained largely unchanged, indicating that age and sex did not substantially account for the differences in time-to-RTW between the various samples.

### Predictors of time-to-RTW

Respondents who did not provide a response to all questions included in the CPH models were excluded (N=225), leaving 30 171 observations included (see table 1 for full details of the sample). To evaluate the contribution of various predictors to time-to-RTW, first univariate CPH models were fitted to the data (table 3). All predictors were significantly associated to time-to-RTW, P<0.001. All significant predictors were associated with later RTW, except for mild rhinitis and sore throat, which were related to earlier RTW.

**Table 3:** Prediction of return to work (RTW): output of univariate and multivariate Cox proportional hazards (CPH) models. [HR=hazard ratio; CI=confidence interval].

Variable	Predictors of time to RTW (N=30 171)		Extended set of predictors of time to RTW (N=8 502) ^a^	
	
	HR	95% CI	HR	95% CI
Univariate CPH models				
Age (years)	0.98	0.98–0.99	0.99	0.99–0.99
Sex (female)	0.87	0.85–0.89	0.87	0.83–0.91
Disease burden	0.90	0.90–0.90	0.90	0.89–0.91
Risk group				
Yes	0.70	0.67–0.73	0.77	0.72–0.82
Perhaps/somewhat	0.78	0.75–0.82	0.92	0.86–0.99
Symptoms				
Fatigue	0.60	0.59–0.62	0.62	0.58–0.66
Headache	0.88	0.86–0.90	0.98	0.93–1.04
Coughing	0.96	0.93–0.98	1.16	1.10–1.22
Mild rhinitis	1.04	1.01–1.06	1.21	1.16–1.27
Muscle pain	0.80	0.78–0.82	0.92	0.88–0.96
Fever	0.89	0.87–0.91	0.98	0.93–1.02
Sore throat	1.15	1.13–1.18	1.20	1.15–1.26
Shortness of breath	0.63	0.62–0.65	0.68	0.65–0.71
Coughing up mucus	0.92	0.90–0.95	0.97	0.93–1.01
Nausea/vomiting	0.73	0.70–0.75	0.82	0.78–0.87
Intestinal problems	0.74	0.71–0.76	0.82	0.77–0.87
Loss of taste			0.84	0.80–0.88
Job satisfaction			0.99	0.97–1.01
Sleep disturbance			0.92	0.90–0.94
Multivariate CPH model				
Age (years)	0.99	0.99–0.99	0.99	0.99–0.99
Sex (female)	0.88	0.86–0.90	0.87	0.83–0.91
Risk group				
Yes	0.81	0.77–0.84	0.83	0.77–0.89
Perhaps/somewhat	0.85	0.82–0.89	0.96	0.90–1.03
Symptoms				
Fatigue	0.70	0.68–0.72	0.67	0.63–0.72
Headache	0.98	0.95–1.00	1.01	0.95–1.07
Coughing	1.07	1.04–1.10	1.24	1.17–1.32
Mild rhinitis	1.08	1.06–1.11	1.23	1.17–1.29
Muscle pain	0.90	0.88–0.92	0.97	0.92–1.02
Fever	1.00	0.98–1.03	1.00	0.96–1.05
Sore throat	1.24	1.21–1.27	1.19	1.14–1.25
Shortness of breath	0.70	0.68–0.72	0.72	0.69–0.76
Coughing up mucus	1.00	0.97–1.02	0.99	0.94–1.04
Nausea/vomiting	0.83	0.81–0.86	0.90	0.85–0.96
Intestinal problems	0.87	0.84–0.90	0.90	0.85–0.96
Loss of taste			0.86	0.82–0.91
Job satisfaction			1.00	0.98–1.02
Sleep disturbance			0.95	0.92–0.97

a Variables in the “predictors of time to RTW” model plus loss of taste as a symptom, as well as job satisfaction and sleep disturbance.

Next, a multivariate CPH model was fit (concordance index=0.63; table 3). Age contributed to time-to-RTW (HR 0.99, 95% CI 0.99–0.99), as well as sex, with later RTW for females (HR 0.88, 95% CI 0.86–0.90). Persons belonging to a risk group (HR 0.81, 95% CI 0.77–0.84) or indicating possibly belonging to a risk group (HR 0.85, 95% CI 0.82–0.89) also showed delayed RTW. The symptoms most strongly related to time-to-RTW were fatigue (HR 0.70, 95% CI 0.68–0.72) and shortness of breath (HR 0.70, 95% CI 0.68–0.72). The presence of these symptoms delayed RTW. The symptom sore throat (HR 1.24, 95% CI 1.21–1.27) was most strongly associated with earlier RTW.

### Predictors of time-to-RTW: extended set of predictors

In addition to the predictors of the first CPH models, the second CPH models also included the predictor loss of taste as a symptom, as well as job satisfaction and sleep disturbance. The data entered in this second CPH model comprised only responses of employees with first day of absence between 25 January 2021 and 18 March 2022 and a self-estimated time-to-RTW of more than a week (N=8502; table 1). With this smaller dataset, the univariate CPH models showed that headache, fever and coughing up mucus were no longer associated to RTW (table 3). The newly added predictors loss of taste (HR 0.84, 95% CI 0.80–0.88) and sleep disturbance (HR 0.92 95% CI 0.90–0.94) were univariately associated with later RTW for persons with sleep disturbance. Job satisfaction (HR 0.99, 95% CI 0.97–1.01) was not univariately associated with RTW.

Next, a multivariate CPH model was fit (concordance index=0.62; table 3). Compared to the first multivariate CPH model, some predictors no longer contributed significantly (risk group (perhaps/somewhat), headache, muscle pain, and fever). The newly added symptom loss of taste was associated to later RTW (HR 0.86, 95% CI 0.82–0.91). Job satisfaction was unrelated to RTW (HR 1.00, 95% CI 0.98–1.02), while sleep disturbance was associated with later RTW (HR 0.95, 95% CI 0.92–0.97).

## Discussion

This study shows that RTW after COVID-19 was notably later than after flu-like symptoms but became earlier over successive periods with different dominant virus variants. The main predictors contributing to later RTW were older age, female sex, belonging to a risk group, and the symptoms fatigue and shortness-of-breath.

### COVID-19 and return to work

The results show that, in general, COVID-19 clearly surpasses flu-like conditions in terms of sickness duration (median time-to-RTW=10 versus 6 days, respectively). Nonetheless, RTW became earlier with each new variant: median time-to-RTW was 15 days in the alpha-dominant period and shortened to 12 days in the delta-dominant period and to 7 days in the omicron-dominant period. In fact, the RTW rate after COVID-19 in the last period seems to approximate the RTW rate after flu-like conditions. On average, 90.6% of employees returned to work within 6 weeks and 97.0% within 6 months, resembling findings from a Danish cohort (5). Of the employees who contracted COVID-19, 5.5% were absent for >12 weeks. This number was 12.8% in the alpha-dominant period but declined to 5.8% in the delta-dominant period and to 1.4% in the omicron-dominant period.

There may be several reasons why time-to-RTW shortened with successive virus variants. It is known that particularly for omicron, transmission rate is higher but disease severity is less (13, 14) – although the latter may be conflated with increased vaccination rates while omicron was attaining dominance. The decrease in the proportion of long-term absenteeism in the omicron-dominant period must therefore be set against a sharp increase in the total number of infections and the number of short-term sicknesses. Other factors that may explain variations in RTW over the course of the COVID-19 pandemic are behavioral adjustments, compliance with government measures (eg, working from home), better adapted healthcare, or a shift in infected population to less vulnerable employees.

Given the massive scale of the COVID-19 pandemic, a relatively small share of long-term absence after COVID-19 still represents a major burden to public health and the economy (15, 16). As such it may have disruptive effects on society, for example when important functions and services can no longer be provided due to long-term sickness absence of staff (eg, front line healthcare workers). Persistent symptoms of COVID may also have a long lasting impact on the workability of employees. They may force individuals to work reduced hours and ultimately increase the risk of unemployment and financial hardship (15–17).

In the present study, we assessed absence duration rather than persistence of symptoms after COVID-19. Employees may have returned to work while still suffering from post-infection sequelae causing functional or neurological impairment and presenteeism (17–19). A recent meta-analysis shows that a significant proportion of patients experience persistent fatigue and/or cognitive impairment in the period following COVID-19 (20). For these reasons, and to spare employees long and frustrating recovery processes, it remains vital that employers put continued effort into preventing COVID-19 contraction at work as well as mitigating the negative impact of COVID-19. This can be achieved by fostering a long-term COVID-19 safety climate (21) or through comprehensive organizational interventions (22). Furthermore, employers should be aware that a significant number of employees with COVID-19 may experience long-term symptoms, and have systems in place to facilitate sustainable return to work for these employees (23).

### Predictors of return to work

Although more reports are emerging (5–8), it is still unclear why persons with seemingly similar disease profiles have diverging recovery trajectories after COVID-19 (see eg, 15). The present study confirms that older age and female sex contribute to later RTW after COVID-19 (5–8). One reason for sex differences in RTW might be that females more often have occupations that allow for less flexibility (eg, working from home), although female sex as well as age are also associated to delayed recovery after COVID-19 in general (ie, outside the work context; 24–29). This effect could be most pronounced in healthcare staff during the earliest phases of the pandemic. Comorbidity has also been identified as a limiting factor for RTW (5, 26), which is in line with our finding that employees belonging to a risk group are prone to later RTW.

Although some data show that hospitalization is a risk factor for protracted illness (5, 6, 29), it has also been argued that symptom severity in the acute phase is not always predictive of the duration of the illness (15). While disease burden was dropped from multivariate analyses, the current univariate results suggest that self-reported disease burden at the time of reporting sick can be an informative predictor of time-to-RTW. This is in line with the finding that number of symptoms is a predictor of disease duration (24). Regarding those symptoms, shortness-of-breath and fatigue were found to be most strongly associated to later RTW in the current study. Although comparison with other studies is difficult due to methodological differences and variations in measured symptoms, these rather generic symptoms have been repeatedly identified as predictors of disease duration (24, 27, 29, 30). Identification of such prognostic factors may aid healthcare professionals in getting a grip on variations in the disease course and the rehabilitation process.

One reason why exact prediction of disease duration and RTW after COVID-19 is difficult may be that, in addition to symptoms in the acute phase and objective factors such as age and sex, psychosocial factors may also play a role. For this reason, we ran an additional analysis on a subset of the data that included the non-medical predictors job satisfaction and sleep disturbance. Results showed that sleep disturbance but not job satisfaction was associated with later RTW. It remains speculative what the origin of these sleeping problems is and whether they are causally related to RTW, however, prevalence of sleep problems in COVID-19 patients was found to be very high (31). As sleep is often considered a key indicator for general health, sleeping problems could be a by-product of physical complaints as well as the result of psychosocial issues. In any case, it is conceivable that for a better understanding of RTW after COVID-19, psychosocial factors such as anxiety (30, 32), stress (8, 32–34), coping and locus of control should be taken into account.

The first multivariate model showed that some of the predictors were associated with an earlier RTW (sore throat, coughing, and mild rhinitis). Of these, the effect of coughing is difficult to interpret as coughing up mucus was also a predictor in the model, and it is unknown how participants weighted these two symptoms against each other. Nevertheless, it remains speculative why some rather mild symptoms were associated with earlier RTW. Reasons might be sought in a complex interaction due to co-occurrence of these symptoms with other more serious symptoms. Also, part of the sample consisted of employees with COVID-19 but without symptoms. These employees may have reported sick only because they had no opportunities to work from home. If that is the case, their time-to-RTW has been artificially extended to the imposed quarantine duration. This group without symptoms may have returned to work later than employees with mild symptoms such as sore throat and mild rhinitis, because the latter group is also partly composed of employees who returned to working from home before their quarantine was over. Finally, the prediction models with the extended set of predictors were created with a subset of the original sample that reported a self-estimated absence duration of at least one week. Although this model was not targeted at estimating absolute RTW rates, this may have introduced a bias to more severe cases.

### Monitoring function of occupational health services

A pandemic is a highly volatile time period in which there is a great need for explanation of the current, and prediction of the future situation. For example, employers want to know what absence rates to anticipate and government agencies want to know how many people will apply for benefits. Referencing RTW rates after COVID-19 with RTW rates after flu-like conditions can aid employers and policymakers in estimating the human and economic burden of COVID-19. Given that the RTW rate varied over the course of the pandemic, it is vital that such figures are based on actual and preferably large volumes of data.

Indeed, in times of a pandemic, rapid and accurate data collection is essential. At the beginning of the COVID-19 pandemic, this was a challenge as virus tests were not readily available. Also, as the pandemic subsided and the number of nationally registered tests declined in favor of self-testing, it became difficult to monitor the evolution of the infection rate and sickness duration. It is therefore vital to exploit other data resources. With flexible and prompt adaptation of an existing survey, the OHS in the current study has gained valuable and current insights into absence rates and absence duration of employees with COVID-19 in The Netherlands. By providing a reliable and constant flow of information, OHS can enable early pandemic monitoring and thus assist policymakers in their decision-making. When data is obtained from a worker population, this may require some extrapolation to the general population – even with a decent and unbiased nationwide coverage of the OHS – but with appropriate weighting factors this could be promising.

### Methodological considerations

The data for the present study were partly obtained through self-reports. For example, COVID-19 status was measured by asking participants for a positive COVID-19 test or a COVID-19 diagnosis confirmed by a doctor or Municipal Health Service. This leaves opportunities for user error, and employees who were awaiting the test result or did not want to be tested were not included. Also, at the start of the pandemic, tests were not readily available, which implies that early infected employees are underrepresented in the total sample. This may have affected RTW estimates for the whole COVID-19 sample. Extrapolating the findings from the comparison of virus variants, it is likely that employees that contracted COVID-19 at the start of the pandemic returned to work later, possibly resulting in underestimated RTW rates. Also, it cannot be ruled out that employees with ordinary colds also entered the reference group, as the symptoms of colds and flu are sometimes hard to distinguish. This may have biased estimates for the reference group towards earlier RTW.

Differences in test availability are not an issue for the three virus-variant samples, as the alpha-dominant time period started well after large-scale testing was organized in The Netherlands. However, for these three subsamples, length of follow-up was logically different, making it difficult to draw conclusions on RTW for long absence cases (eg, more than six months or even a year), in particular for the omicron-dominant period.

Employees from sectors that accommodate working from home may also been underrepresented in the sample as they are less likely to report sick with symptoms if working from home is still feasible. It is also possible that employees who were sick for a short period did not complete the survey, for example when they received it after they had already returned to work. This could have led to an overestimation of time-to-RTW. Conversely, severely ill employees may have been too sick to complete the survey, which may have resulted in smaller estimates of time-to-RTW. On the other hand, quarantine measures may have artificially extended time-to-RTW in cases where working from home was not an option.

Finally, large companies were underrepresented in the current study, which may have affected RTW estimates if employees of smaller companies have different absence trajectories than those working in larger companies.

## Supplementary material

Supplementary material
