# Supplementary material for: Return-to-work rates and predictors of absence duration after COVID-19 over the course of the pandemic
Source: Scand J Work Environ Health. 2023 Mar 30;49(3):182–92. doi: 10.5271/sjweh.4077 (PMC10621901; doi:10.5271/sjweh.4077)
Supplement: Supplementary material [file SJWEH-49-182-S001.pdf]

Return-to-work rates and predictors of absence duration after COVID-19 over the course of the pandemic[1]

by Bart Aben, PhD,[2] Robin N Kok, PhD, Astrid de Wind, PhD

1. Supplementary material
2. Correspondence to: Bart Aben, PhD, HumanTotalCare B.V., Department of Research and Business Development, Zwarte Woud 10, 3524 SJ Utrecht, The Netherlands. [E-mail: b.aben@humantotalcare.nl]

Figure S1. *Questions in triage-survey ArboNed*.

Questions in triage-survey ArboNed

What is the reason for your absence?<br>*If you have multiple health complaints, please choose the group of complaints that represent the primary reason for your absence from work.*	:	☐ 1. Corona<br>*(Also choose this option if you have a suspicion)*<br>☐ 2. Influenza or flu-like complaints<br>☐ 3. Physical health complaints<br>*(For example: injuries, cardiovascular diseases or complaints to your airways or abdomen)*<br>☐ 4. Mental health and/or stress-related health complaints<br>☐ 5. A combination of physical and mental health/stress-related health complaints<br>☐ 6. Complaints related to pregnancy or childbirth<br>☐ 7. I am waiting for an operation<br>*(Not applicable if you suffer from physical complaints)*<br>☐ 8. I am recovering from an operation<br>☐ 9. Other health complaints, namely ____________

Questions: coronavirus (COVID-19)

The answers to the following questions are processed with full regard for privacy law and legislation and are not shared with your employer. If your notification of illness is connected with the COVID-19 coronavirus, it is treated in accordance with our normal rules for notifications of illness.

Note: if your health condition worsens, we advise you to telephone your GP. They will assess your risk of COVID-19 infection and, if necessary, contact the GGD on your behalf.

Your anonymized answers to the following questions can be used to help research into the effects of the COVID-19 coronavirus on sickness absence. Do you consent to this?	:	☐ 1. Yes, I consent to this<br>☐ 2. No, I do not consent to this
Do you think you are infected with the COVID-19 coronavirus?	:	☐ 1. Yes, definitely<br>☐ 2. Yes, probably<br>☐ 3. Probably not<br>☐ 4. Definitely not

Has this been medically confirmed? :

- ☐ 1. No
- ☐ 2. Yes, my GP, doctor or the Municipal Health Services have told me that I probably have been infected
- ☐ 3. I'm waiting for the results of the test
- ☐ 4. I've been tested for COVID-19 coronavirus and the test confirmed that I DO have it
- ☐ 5. I've been tested for COVID-19 coronavirus and the test confirmed that I do NOT have it

---

Do you have any of the following symptoms? :
Select the symptoms that you have experienced

- ☐ 1. No, I have none of these symptoms
- ☐ 2. Fever
- ☐ 3. Coughing
- ☐ 4. Fatigue
- ☐ 5. Coughing up mucus
- ☐ 6. Shortness of breath
- ☐ 7. Muscle pains
- ☐ 8. Sore throat
- ☐ 9. Headache
- ☐ 10. Nausea / vomiting
- ☐ 11. Mild rhinitis
- ☐ 12. Intestinal complaints
- ☐ 13. Loss of taste *(added on January 25, 2021)*
- ☐ 14. Other, namely ____________

How much do you suffer from these symptoms, on a scale of 1 (no suffering) to 10 (a great deal of suffering?) :

- ☐ 1. 1
- ☐ 2. 2
- ☐ 3. 3
- ☐ 4. 4
- ☐ 5. 5
- ☐ 6. 6
- ☐ 7. 7
- ☐ 8. 8
- ☐ 9. 9
- ☐ 10. 10

Are you in a COVID-19 risk group? :

- ☐ 1. Yes
- ☐ 2. Perhaps / somewhat
- ☐ 3. No

On what grounds are you in a risk group? :
More than one answer may be given

- ☐ 1. Age
- ☐ 2. Chronic illness
- ☐ 3. Pulmonary diseases (asthma, COPD)
- ☐ 4. Compromised immune system due to disease and/or medication
- ☐ 5. Smoking
- ☐ 6. Cardiovascular disease
- ☐ 7. Diabetes
- ☐ 8. Obesity
- ☐ 9. Other, namely _____________

Additional questions

I really like my work :

- ☐ 1. No, never
- ☐ 2. No, nearly never
- ☐ 3. Yes, now and then
- ☐ 4. Yes, regularly
- ☐ 5. Yes, often
- ☐ 6. Yes, very often
- ☐ 7. Yes, always

Have you had trouble falling asleep in the past month? :

- ☐ 1. Never
- ☐ 2. Sometimes
- ☐ 3. Regularly
- ☐ 4. Often
- ☐ 5. Always

Supplementary table S1. *Characteristics of the participants included in the sample with flu-like symptoms and the samples with different dominant virus types.*

**Characteristics**	**Flu-like symptoms**			**COVID-19**								
				**Alpha dominant**			**Delta dominant**			**Omicron dominant**		
	n	%	M (SD)	n	%	M (SD)	n	%	M (SD)	n	%	M (SD)
Total	15 862	100		3651	100		5628	100		15 863	100	
Age (years)			40.7 (12.3)			41.9 (11.9)			40.2 (12.1)			40.6 (12.3)
Sex:												
male	9068	57.2		1929	52.8		2890	51.4		9068	57.2	
female	6790	42.8		1722	47.2		2735	48.6		6791	42.8	
prefer not to say	4	0.0		0	0.0		3	0.1		4	0.0	
Disease burden (1 to 10)						6.4 (2.4)			6.3 (2.4)			6.1 (2.4)
Risk group:												
no				2883	79.4		4571	81.9		12 342	77.8	
perhaps/somewhat				382	10.5		509	9.1		1349	8.5	
yes*:				366	10.1		499	8.9		1279	8.1	
respiratory disorder (asthma, COPD)				253	34.3		370	37.1		994	38.4	
age				203	27.5		209	21.0		418	16.1	
chronical disease				98	13.3		162	16.2		409	15.8	
cardiovascular disease				94	12.7		118	11.8		333	12.9	
diabetes				85	11.5		94	9.4		229	8.8	
obesity				88	11.9		99	9.9		250	9.6	
compromised immune system due to disease and/or medication				46	6.2		77	7.7		233	9.0	
smoking				40	5.4		36	3.6		147	5.7	
unknown				20	0.0		49	0.0		893	5.6	
Symptoms*:												
fatigue				2907	80.1		4265	76.4		10 849	72.5	
coughing				2546	70.1		4174	74.8		11 382	76.0	
headache				2524	69.5		3981	71.4		10 826	72.3	
mild rhinitis				2020	55.6		3524	63.2		9030	60.3	
muscle pain				2194	60.4		3082	55.2		8090	54.0	
sore throat				1444	39.8		2323	41.6		9082	60.7	
fever				1709	47.1		2834	50.8		7345	49.1	
coughing up mucus				1133	31.2		1991	35.7		5834	39.0	
shortness of breath				1352	37.2		1827	32.7		4457	29.8	
loss of taste				1363	37.5		2641	47.3		2409	16.1	
nausea/vomiting				592	16.3		830	14.9		1985	13.3	

intestinal problems	495	13.6		704	12.6		1674	11.2	
no complaints	106	2.9		133	2.4		404	2.7	
unknown	20	0.0		49	0.0		893	5.6	
Job satisfaction**			5.9 (1.1)			5.8 (1.1)			5.8 (1.1)
Sleep disturbance**			1.8 (0.9)			1.9 (0.9)			2.0 (0.9)

* Multiple answers possible. ** This question was answered only by employees with first day of absence between January 25, 2021 and March 18, 2022 and a self-estimated RTW of more than a week (n =2107 , n =, and n = 2564 for the alpha, delta, and omicron-dominant samples, respectively

Supplementary table S2. *Probability of returning to work after COVID-19 at various RTW rates and in time periods with different dominant virus variants after propensity score matching on age and sex. The sample for flu-like symptoms and the total COVID-19 sample include observations with first day of absence between March 19, 2020 and March 18, 2022. The other samples include observation with first day of absence between February 15, 2021 and June 27, 2021 (alpha dominant), June 28, 2021 and December 27, 2021 (delta dominant), and December 28, 2021 and March 18, 2022 (omicron dominant). The sample of participants with flu-like complaints was matched to the total sample of COVID-19 participants. The delta- and omicron-dominant samples were matched to the alpha-dominant sample.*

	**Flu-like symptoms**				**COVID-19**															
					**Total**				**Alpha dominant**				**Delta dominant**				**Omicron dominant**			
	**n**	**M (SD)**	**% (95% CI)**	**Mdn (95% CI)**	**n**	**M (SD)**	**% (95% CI)**	**Mdn (95% CI)**	**n**	**M (SD)**	**% (95% CI)**	**Mdn (95% CI)**	**n**	**M (SD)**	**% (95% CI)**	**Mdn (95% CI)**	**n**	**M (SD)**	**% (95% CI)**	**Mdn (95% CI)**
Total	15 589				15 589				3651				3651				3651			
Age		40.7 (12.3)				40.8 (12.4)				41.9 (11.9)				42.0 (12.3)				42.1 (12.6)		
Sex																				
Male			57.2				57.1				52.8				51.1				50.5	
Female			42.8				42.8				47.2				49.9				49.5	
Time to RTW in days				6 (6-6)				12 (12-12)				15 (14-15)	12			12 (12-12)				7 (7-8)
RTW																				
≤ 1 week			71.6 (70.9-72.3)				26.8 (26.1-27.4)				14.0 (12.9-15.2)				24.3 (23.0-25.8)				49.5 (47.9-51.1)	
≤ 3 weeks			91.9 (91.4-92.3)				64.4 (63.6-65.1)				48.5 (46.9-50.1)				66.6 (65.1-68.2)				86.6 (85.5-87.7)	
≤ 6 weeks			99.2 (99.0-99.4)				87.9 (87.4-88.4)				79.2 (77.9-80.5)				89.1 (88.0-90.1)				97.0 (96.4-97.5)	
≤ 12 weeks			100.0 (100.0-100.0)				92.8 (92.4-93.2)				87.2 (86.0-88.2)				94.0 (93.2-94.8)				98.3 (97.7-98.7)	
≤ 6 months			100.0 (100.0-100.0)				98.2 (98.0-98.4)				92.9 (92.0-93.7)				97.0 (96.3-97.5)				*	

* There are no omicron-infected employees with RTW after six months at the time of writing this report. CI=confidence interval.

The survival rate differed between the total COVID-19 sample and the sample with flu-like complaints, $\chi^2(1)$=7398.03, P<0.001. Multi-variate log-rank tests also showed a difference between the three survival distributions, $\chi^2(2)$ =1758.47, P<0.001. Bonferroni-corrected pairwise comparisons showed differences between all three distributions (P*'s* <0.001).

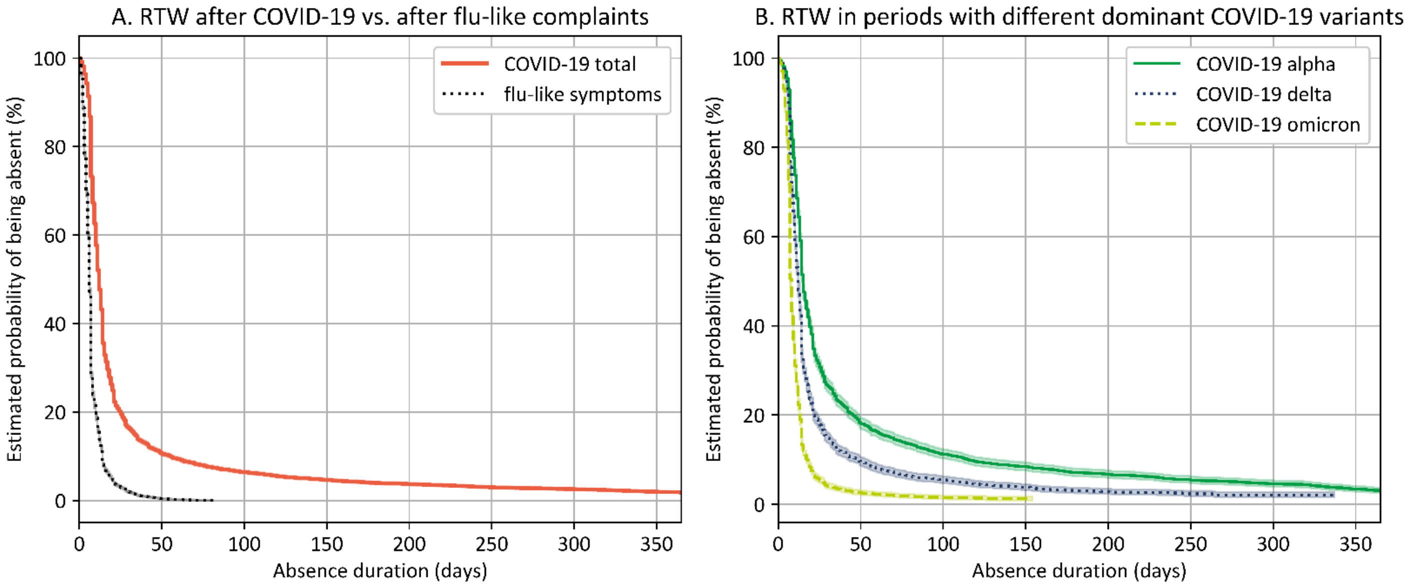

Supplementary figure S2. Survival curves after propensity score matching on age and sex. A) Survival curve of employees with self-reported COVID-19 between March 19, 2020 and March 18, 2022 (Covid-19 total), referenced against employees with self-reported flu-like symptoms in the same time period. B) Survival curves of employees with self-reported COVID-19 in time periods with different dominant variant are also displayed. Information on variant dominance was taken from the Dutch National Institute for Public Health and the Environment (RIVM; https://coronadashboard.government.nl/landelijk/varianten). Error bands represent exponential Greenwood confidence intervals.
